# A comprehensive investigation of biochemical status in patients with telogen effluvium: Analysis of Hb, ferritin, vitamin B12, vitamin D, thyroid function tests, zinc, copper, biotin, and selenium levels

**DOI:** 10.1111/jocd.16512

**Published:** 2024-08-06

**Authors:** Irem Nur Durusu Turkoglu, Aziz Kaan Turkoglu, Seçil Soylu, Gülcan Gencer, Rümeysa Duman

**Affiliations:** ^1^ Department of Dermatology Afyonkarahisar Health Sciences University Afyonkarahisar Turkey; ^2^ Internal Medicine Clinic Afyonkarahisar State Hospital Afyonkarahisar Turkey; ^3^ Department of Biostatistics Afyonkarahisar Health Sciences University Afyonkarahisar Turkey; ^4^ Department of Biochemistry Afyonkarahisar Health Sciences University Afyonkarahisar Turkey

**Keywords:** biotin, copper/zinc, selenium, vitamin D, zinc

## Abstract

**Aim:**

The etiology of telogen effluvium (TE) includes situations that may cause physiological stress, surgical trauma, inflammatory, infectious, iatrogenic causes, medications and nutritional deficiencies. TE has been associated with iron deficiency, vitamin B12 deficiency and thyroid diseases. In recent years, the use of over‐the‐counter food supplements containing vitamins and minerals such as biotin, vitamin D, zinc (Zn), copper (Cu) and selenium (Se) has been increasing in TE patients. The aim of this study is to investigate whether there are differences in nutritional status, vitamin and mineral levels by comparing individuals with TE and a control group.

**Materials and Methods:**

This case–control study included 90 female patients diagnosed with chronic telogen effluvium (CTE), and 90 female controls volunteered to participate in the study who consulted for reasons other than TE. Both groups aged 18 and over and applied to dermatology polyclinic between 01.09.2022 and 01.09.2023. A detailed anamnesis was taken from all patients, a hair pull test was performed, and TE was diagnosed after a dermoscopic examination was performed on all areas of the scalp. Then, serum vitamin D, Zn, Cu, Se levels and biotin levels in serum and urine were measured. Hemoglobin (Hb), ferritin, vitamin B12 and thyroid function tests were retrospectively scanned from the hospital database.

**Results:**

It was determined that Zn levels were significantly lower in CTE patients than in controls. Se levels were found to be significantly higher in patients than in controls. There was no difference in Hb, ferritin, vitamin B12, thyroid function tests, vitamin D, Cu levels, serum and urine biotin levels between the two groups. Zn, Cu/Zn and Se levels were found to have statistically significant diagnostic performance in predicting the diagnosis of CTE. Cu/Zn ratio and Se value were found to be significant predictors of CTE.

**Conclusion:**

This study shows us that nutritional deficiencies are not as common as thought in patients diagnosed with TE. Other causes that may cause TE should be investigated by a detailed anamnesis and a good physical examination. After all, tests for suspected conditions should be performed and individualized treatment options should be created for each patient.

## 1 INTRODUCTION

Throughout generations, strong, thick, and shiny hair has been associated with good health and attractiveness. Hair loss (alopecia) not only indicates potential systemic diseases but also significantly affects the quality of life by impacting beauty and self‐image. Telogen effluvium (TE) was first described in 1961 by Albert Kligman as a result of physiological stress causing accelerated transition from the anagen to early telogen phase after a stressful event.^1^ This ultimately leads to significant hair loss about 2–3 months later, prompting many patients to seek medical help. If TE lasts less than six months it is referred to as acute TE (ATE); durations longer than that are known as chronic TE (CTE).^2^

Many factors can disrupt the normal hair cycle, including medications, physiological stress conditions such as surgical trauma, bleeding, high fever, chronic systemic diseases, pregnancy, emotional stress, hypo‐hyperthyroidism, connective tissue diseases, hematological malignancies, hepatic failure, renal failure, inflammatory bowel diseases, and chronic infectious processes.^3^

Severe calorie restrictive diets and prolonged fasting, iron deficiency, zinc (Zn) deficiency, and essential fatty acid deficiencies are among the most well‐known dietary deficiencies. Due to the important functions of vitamin D in cell growth, it is thought that vitamin D deficiency may also cause hair loss.^4^

The amount and quality of hair heavily relies on an individual's nutritional status. Cells with high biosynthetic activity, like hair follicles, require a sufficient supply of proteins, trace elements, and vitamins for optimal functioning. In cases of inadequate protein‐calorie intake or deficiencies in trace elements and vitamins, there is an inevitable deterioration in hair growth and skin structure.

However, the current trend involves widespread consumption of supplements containing essential vitamins and minerals without properly assessing the presence or absence of nutritional deficiencies. Biotin, Zn, copper (Cu), selenium (Se), iron, vitamin B12, and vitamin D are among the frequently included components in these supplements. There is a debate about whether increasing the content of an already sufficient diet with additional supplements will further enhance hair growth. Our study aimed to examine differences in hemoglobin, ferritin, vitamin, and mineral levels between TE patients and healthy controls.

## 2 MATERIALS AND METHODS

This study was designed as a case–control study and involved 90 patients diagnosed with CTE, alongside 90 controls who applied to the dermatology outpatient clinic. The patient group comprised females aged 18 and above, who had experienced hair loss for at least 6 months, were clinically and dermoscopically diagnosed with CTE, and willingly took part in the research. The control group comprises female volunteers who applied for reasons other than inflammatory disease, did not report hair loss, and had their diagnosis of TE ruled out by dermatological examination. Participants with other hair diseases, hepatic or renal disorders, recent consumption of tested vitamin and mineral containing supplements, broad‐spectrum antibiotics or chemotherapy, as well as those who were pregnant or lactating were excluded from the study.

Dermatological examinations were conducted on the participants to assess hair density, quantity, and quality. To rule out other potential hair‐related diseases, the scalp underwent a detailed examination, all patients received a hair pull test, and trichoscopic examination was applied. Patients who had been experiencing hair loss for more than six months, with over 10% hair loss in all areas as determined by the hair pull test and no other visible signs of a specific hair disease, were diagnosed with CTE.

The blood samples taken from the participants during their routine admission were stored for 30 min, then centrifuged at 2000 ×*g* at +4 C for 10 min to separate the serum portion. Ferritin, vitamin B12, TSH, and fT4 levels were measured. The remaining serum samples were stored at −80°C for biotin, Zn, Cu, Se, and vitamin D measurements. Blood was collected into tubes containing K3EDTA for the complete blood count. The urine samples were stored at −80°C for biotin measurement.

Serum vitamin D and biotin, as well as urine biotin, were measured using ELISA kits from BT‐LAB. The absorbance readings were taken with the ChemWell 2910 ELISA reader and the results were reported in ng/ml. Serum Se measurement was carried out using a PerkinElmer ICP‐MS device in helium KED mode. A volume of 250 μL from the sample was combined with 5 mL of diluent for completion.

Serum Cu and Zn levels were measured using the Cu and Zn measurement kits from Rel Assay Diagnostics. The absorbance readings were obtained with the ChemWell 2910 ELISA reader, and the results were reported in μg/dL.

The data was analyzed using IBM SPSS Statistics for Windows version 26.0. Normality was assessed using the Shapiro–Wilk test, and the data are presented as mean ± standard deviation. For independent pairwise comparisons, Student's *t*–test was used, while ANOVA test was employed for comparisons involving more than two normally distributed groups. In cases of non‐normal distribution, Mann–Whitney *U* test and Kruskal–Wallis *H* test were utilized for independent pairwise comparisons and comparisons with more than two groups, respectively.

Categorical variables were represented as numbers (%) and assessed using the Chi‐Square test. Spearman correlation was employed for the correlation analysis, while Roc analysis was conducted to evaluate diagnostic tests. Logistic regression was utilized to assess risk factors. The statistical significance level for all tests performed was set at *p* < 0.05. A sample size of 88, determined by the trial version of G power 3.1.9.4 package program, resulted in the inclusion of 90 patients and 90 controls in the study.

## 3 RESULTS

The mean age of the patient group was 28.52 ± 9.57, while that of the control group was 28.07 ± 9.61. Table 1 presents the age, height, weight, and BMI values for the patient and control groups, while Table 2 details their respective socioeconomic statuses.

**TABLE 1:** Age, weight (kg), height (cm), BMI in the patient and control groups.

Variables	Patient				Control			
	Min.	Max.	Mean	SD	Min.	Max.	Mean	SD
Age	18	58	28.52	9.57	18	59	28.07	9.61
Weight	44	106	63.18	12.81	45	102	64.67	12.85
Height	150	180	162.64	5.86	145	178	163.15	7.13
BMI	17.01	36.68	23.84	4.34	15.92	39.84	24.35	4.91

**TABLE 2:** Demographic variables in the patient and control groups.

Variables	Patient		Control	
	*N*	Percentage	*N*	Percentage
Smoking				
No	69	76.7	71	78.9
Yes	21	23.3	19	21.1
Alcohol consumption				
No	85	94.4	83	92.2
Yes	5	5.6	7	7.8
Socioeconomic status				
Low	44	48.9	41	45.6
Medium	39	43.3	41	45.6
High	7	7.8	8	8.9

The most common dermoscopic findings used in the diagnosis of TE were a decrease in hair density (98.8%) and empty follicles (98.8%). This was followed by an increase in the follicular unit with a single hair follicle (91.1%), newly growing short and normal hair (84.4%), and yellow dots (62.2%) (Table 3).

**TABLE 3:** Frequency of dermoscopic findings in the patient group.

Dermoscopic findings	*N*	Percentage
Decrease in hair density	89	98.8
Empty follicles	89	98.8
Yellow dots	56	62.2
Increase in the follicular unit with a single hair follicle	82	91.1
Newly growing short and normal hair	76	84.4

When comparing the biochemical values (Table 4) of patients with TE to those of the control group, it was found that the mean Hb value was 13.25 g/dL in the patient group and 13.04 g/dL in the control group. The mean ferritin value was 45.60 μg/L in the patient group and 30.55 μg/L in the control group. Although the ferritin value was lower in the control group compared to the patient group, this difference was not statistically significant (*p* = 0.508). Iron deficiency was detected in 12.2% of the patient group and 16.6% of the control group, with no statistically significant difference between them.

**TABLE 4:** Comparison of biochemical results in patient and control groups.

Variables	Patient	Control	*p*‐value
Vit‐D (ng/mL)	17.57 ± 7.44	19.5 ± 8.74	0.206^b^
Cu (μg/dL)	139.99 ± 45.07	136.16 ± 32.56	0.656^b^
Zn (μg/dL)	49.91 ± 22.21	60.53 ± 29.27	**0.004** ^b^
Biotin (ng/mL)	86.42 ± 37.15	93.82 ± 39.47	0.324^b^
Urine Biotin (ng/mL)	158.48 ± 39.08	164.96 ± 43.30	0.293^a^
Se (μg/L)	71.76 ± 9.84	66.96 ± 9.33	**0.001** ^a^
Hb (g/dL)	13.25 ± 1.21	13.04 ± 1.30	0.277^b^
Ferritin (μg/L)	45.60 ± 142.11	30.55 ± 27.56	0.508^b^
Vit B12 (ng/L)	352.70 ± 129.45	366.72 ± 127.81	0.231^b^
TSH (mIU/L)	3.05 ± 9.45	2.13 ± 1.21	0.183^b^
St4 (ng/dL)	1.29 ± 0.17	1.29 ± 0.25	0.835^b^

*P* value < 0.05 were written in bold.

^a^ Student's *t* test.

^b^ Mann–Whitney U test.

The mean vitamin B12 value in the patient group and control group was found to be 352.70 and 366.72 ng/L, respectively. Although the vitamin B12 value was lower in the patient group than in the control group, this difference was not significant. Vitamin B12 deficiency was 4.44% in the patient group and 3.33% in the control group, with no significant difference between them.

The mean TSH level was 3.05 mIU/L in the patient group and 2.13 mIU/L in the control; mean fT4 levels were found to be 1.29 ng/dL in the patient group and 1.29 ng/dL in the control and no significant difference was detected between the groups in terms of thyroid function tests.

The mean vitamin D level was found to be 17.57 ng/mL in the patient group and 19.5 ng/mL in the control and no significant difference was detected between them.

Cu levels were found to be 139.99 μg/dL in the patient group and 136.16 μg/dL in the control, no significant difference was detected.

While the mean Zn value in the patient group was 49.91 μg/dL, it was 60.53 μg/dL in the control and the Zn value of the patient group was significantly lower than the control (*p* = 0.004). ROC analysis was carried out to measure the performance of the Zn value in predicting the diagnosis of CTE. It was found that Zn level (AUC: 0.624 and *p* = 0.004) had a statistically significant diagnostic performance in predicting the diagnosis of CTE. The sensitivity and specificity were found to be 67.78% and 55.56%, respectively, for the best cut‐off point of Zn level ≤54.85 μg/dL.

The mean Se concentration in the patient group was found to be 71.76 μg/L, while in the control group it was 66.96 μg/L. Statistical analysis revealed that the Se concentration was significantly higher in the patient group than in the control group (*p* = 0.001). After evaluating the diagnostic performance of Se level in predicting the diagnosis of CTE through ROC analysis, it was observed that Se level had a statistically significant diagnostic performance in predicting the diagnosis of CTE. The sensitivity of the Se level, which was determined for the best cut‐off point of >67.98 μg/L, was 70%, while its specificity was 56.67%.

Both serum biotin (86.42 ng/mL) and urine biotin (158.48 ng/mL) levels in the patient group were higher than serum biotin (93.82 ng/mL) and urine biotin (164.96 ng/mL) levels in the control but this difference was not statistically significant.

A moderate positive correlation was found between vitamin D and biotin in both the patient and control groups. While a low positive correlation was found between Zn and Cu in the control group, this relationship disappeared in the patient group.

When the patient and control groups were compared in terms of Cu/Zn ratio (Table 5), the mean Cu/Zn ratio was found to be 3.42 in the patient group and 2.66 in the control, and the Cu/Zn ratio in the patient group was found to be significantly higher than the control (*p* = 0.009). ROC analysis was performed to measure the performance of the Cu/Zn value in predicting the diagnosis of CTE revealed that the Cu/Zn value (AUC: 0.613 and *p* = 0.007) had a statistically significant diagnostic performance in predicting the diagnosis of CTE. The sensitivity and specificity determined for the best cut‐off point of Cu/Zn >2.79 were found to be 53.33% and 66.67%.

**TABLE 5:** Comparison of Cu/Zn ratio in patient and control groups.

Variable	Group	N	Mean	SD	*p*‐value^a^
Cu/Zn ratio	Patient	90	34 226	23 588	0.009
	Control	90	26 687	1581	

^a^ Mann–Whitney U test.

Binary multivariate logistic regression analysis was performed to identify risk factors for hair loss and the results indicated that Cu/Zn ratio and Se value were found to be significant predictors of CTE (OR = 0.77, *p* = 0.017; OR = 0.94, *p* = 0.001, respectively).

## 4 DISCUSSION

Healthy, thick, and lustrous hair is linked to good health, attractiveness, and a strong personality, for both men and women. As a result, hair loss has always been a matter of significant concern. Assessing patients with hair loss and providing suitable treatments fall under the primary areas of interest in the field of dermatology.^5^, ^6^

Nowadays, the nutritional status of patients with diffuse hair loss is often examined. Nutritional deficiencies can affect not only hair, but also other skin appendages, organs, and systems, which should be treated when detected. However, in recent years, the use of over‐the‐counter (OTC) oral supplements to treat dermatological conditions, including TE, has become increasingly popular.^7^ When examining the literature, the evidence regarding the safety and effectiveness of OTC supplements is quite insufficient. Standardized doses and outcome measurements are not available, and it is unclear whether supplements have any potential side effects. In our study, we evaluated vitamin D, biotin, Zn, Cu, and Se levels between CTE patients and controls, as well as Hb, ferritin, vitamin B12 levels, and thyroid function tests, which are routinely screened in TE patients.

Kantor et al.^8^ conducted a study to compare the serum Hb and ferritin concentrations of patients with different types of hair loss and those without a hair loss complaint. They found that ferritin levels were significantly lower in patients with alopecia areata (AA) and androgenetic alopecia (AGA) compared to those without hair loss. However, no significant difference was observed in ferritin levels between TE and alopecia totalis/universalis patients and controls. The authors suggested that low ferritin levels may not be detected in patients with TE due to various medications, fever, and rapid weight loss that can cause TE. In the same study, no significant difference was observed in the Hb levels. Similarly, in our study, we also found no significant difference in the Hb levels of CTE patients and the control group.

Based on a study conducted on 194 female patients with widespread telogen hair loss lasting for six months or more, it was found that 12 patients had serum ferritin concentrations equal to or lower than 20 ng/mL. Out of these 12 patients, three were diagnosed with CTE. These patients were given iron supplements for a period of 3–6 months until their serum ferritin concentrations increased above 20 ng/mL. However, there were no observable changes in their hair condition post‐treatment.^9^

According to our study, we found that there was no significant difference in ferritin levels between patients with CTE and the control group. This result was consistent with the findings of other studies mentioned in the literature. Based on this, we can conclude that administering iron supplements to increase ferritin levels might not alleviate the symptoms of CTE, especially if the patient does not have anemia.

Testing for vitamin B12 levels in patients with alopecia is a common practice. However, there have been very few studies that investigated the levels of vitamin B12 in these patients as compared to those in the control group. In our study, we compared the mean vitamin B12 levels in both the patient and control groups and found no significant difference between them.

A comparative study was conducted to assess the levels of vitamin B12 in 317 CTE patients and 327 healthy controls. The mean levels of vitamin B12 were found to be comparable between the two groups. However, a significant difference was noted in the percentage of individuals with lower than normal levels of vitamin B12. Specifically, 11.98% of patients and 6.72% of healthy controls exhibited lower than normal levels of vitamin B12.^10^

Various studies have examined the prevalence of vitamin B12 deficiency in patients with TE. Cheung et al.^11^ found a rate of 2.6%, Avcı et al.^12^ found 3.02%, and Güler Özden et al.^13^ found 2%. In our study, we found a 4.44% rate of vitamin B12 deficiency in the patient group and a 3.33% rate in the control group. However, there was no significant difference between the groups, and our results were consistent with the existing literature on the subject.

There have been numerous studies in the literature that explore the correlation between vitamin D levels and hair diseases. In one particular study, which compared vitamin D levels in 100 TE patients and 150 controls, it was found that the vitamin D level was significantly lower in the patient group (11.16 ± 4.49) than in the control group (18.98 ± 10.65).^14^

Karadağ et al.^15^ found that vitamin D levels were significantly higher in patients with ATE and CTE than in a control group. The authors concluded that higher vitamin D levels are not the cause of hair loss, but rather a compensatory response to hair loss. Telogen hair follicles could increase ultraviolet exposure and indirectly promote vitamin D synthesis in the skin because their undifferentiated melanocytes/melanoblasts lack melanin. In our study, no significant difference was found between the vitamin D levels of the patient and control groups, and it is obvious that there are conflicting results on this issue.

Biotin is a substance that holds significant value in the field of dermatology for the treatment of various conditions. However, the efficacy of biotin supplements in the treatment of dermatological diseases in individuals with normal biotin levels remains uncertain due to limited research. Most studies have been conducted on patients suffering from nail disorders or those with hereditary enzyme deficiencies.^16^, ^17^

In their study, Patel et al.^18^ put forth the proposition that the use of biotin supplementation can offer advantages in the treatment of certain health conditions, such as acquired and hereditary biotin deficiency, brittle nail syndrome, and uncombable hair. However, their findings did not yield sufficient evidence to support the use of biotin supplementation in otherwise healthy individuals, barring these rare conditions.

The study conducted by Rahman et al.^19^ revealed that the serum biotin levels in patients with TE were not significantly different from those in the control group. Both groups exhibited optimal serum biotin levels, and the researchers observed that biotin deficiency is a rare occurrence in the population. Consequently, even though the possibility of toxicity is low, it is not recommended to prescribe biotin supplements for all cases of alopecia that are not confirmed by examination.

In our study, we found no significant difference between the serum biotin levels of the participants. Since the level of biotin in plasma varies significantly throughout the day, we also measured the urine biotin levels of all participants as it is considered a better marker in case of biotin deficiency. Anyhow, we did not find any significant difference in the urine biotin levels between the patients and the control group.

Biotin is a vitamin that does not pose a risk of toxicity even when taken in large amounts. However, a warning has recently been issued by the Food and Drug Administration (FDA) that biotin consumption may significantly affect laboratory tests. This warning was deemed necessary following increasing scientific publications that biotin intake may lead to inaccurate measurement and clinical misinterpretation of laboratory tests, including troponin levels.^20^

Biotin‐streptavidin binding is a common laboratory technique aimed at enhancing test sensitivity. However, the presence of biotin in blood may interfere with biotin‐dependent immunoassays. In a clinical study involving healthy adults, it was found that the consumption of oral biotin (10 mg/day for 7 days) resulted in a false reduction in thyroid‐stimulating hormone, N‐terminal pro‐brain natriuretic peptide, and parathyroid hormone levels. Additionally, it led to an increase in free triiodothyronine levels and caused biochemical Graves disease in euthyroid individuals.^21^

Our study, consistent with existing literature, recommends against the routine prescription of oral biotin supplements for hair loss in the absence of confirmed biotin deficiency. In such cases, serum biotin levels should be determined and underlying etiologies of biotin deficiency should be identified and treated. Current data does not provide sufficient evidence to support the use of biotin supplementation in individuals with CTE. Dermatologists should exercise caution while selecting patients for biotin therapy, given that oral biotin supplements have been known to adversely impact certain laboratory test results.

The skin is the third organ in the human body that contains the most Zn. Zn deficiency has been associated with a wide range of dermatological disorders. Acrodermatitis enteropathica is a rare genetic disease that may result from Zn deficiency. Additionally, infectious diseases, including viral warts, herpes, and leishmaniasis, and inflammatory diseases such as acne vulgaris, rosacea, seborrheic dermatitis, psoriasis, and non‐cicatricial alopecia, have all been linked to the insufficiency of Zn. As a result, Zn supplements have been recommended for treating these conditions.^22^

In a study conducted with a total of 39 female patients diagnosed with CTE and 30 healthy female controls, Zn and Cu levels in serum and hair were compared in both groups. Zn and Cu levels in serum and hair samples taken from the CTE group were found to be significantly lower than the control group.^23^

In a study by Kil et al.,^24^ 312 hair loss patients were divided into four groups: AGA, AA, FPHL, and TE. Their serum Zn and Cu levels were measured along with 30 controls. The mean serum Zn value in all hair loss groups was significantly lower than the control, while serum Cu value did not differ between groups. The proportion of patients with serum Zn concentration below the normal limit was significantly higher in the AA and TE groups. There was no correlation between Zn and Cu for all groups.

In our research, our findings indicate that there was no statistically significant correlation between the levels of Zn and Cu in the patient group. Yet, in the control group, a weak but positive correlation (*r* = 0.21; *p* < 0.05) was observed.

Our research kit has provided a reference range of 60–120 μg/dL for Zn. We found that the serum Zn level in the patient group diagnosed with CTE was significantly lower (49.91 ± 22.21 μg/dL, *p* = 0.004) compared to the control group (60.53 ± 29.27 μg/dL). The significantly lower levels of Zn in CTE patients, as compared to the reference range, was found to be a critical finding in our study. Based on this, we recommend the measurement of Zn levels and the use of Zn supplements in CTE patients. Moreover, our study revealed that Zn level has a noteworthy diagnostic performance in predicting the diagnosis of CTE, with an AUC of 0.624 and a *p*‐value of 0.004. While previous studies have investigated the relationship between TE and Zn by examining Zn levels, their diagnostic value for TE has not been studied. In this context, our study makes a significant contribution to the literature by examining the diagnostic value of Zn levels in predicting TE.

The biomarker Cu/Zn ratio has garnered significant attention as a potential tool for investigating a variety of inflammatory diseases. Specifically, research has been conducted to investigate the importance of this biomarker in alopecia areata, as well as other chronic and acute conditions, such as infectious diseases, cardiovascular diseases, type 1 diabetes, metabolic syndrome, myopia, asthma, thalassemia, sickle cell anemia, autism, attention deficit and hyperactivity disorder.^25^ A study was recently conducted to further explore the potential value of the plasma Cu/Zn ratio as a sensitive biomarker for inflammatory or nutritional changes in the elderly. The results of this study may have significant implications for the early detection and treatment of inflammatory diseases in this population.^26^

The present study here reveals that the mean Cu/Zn ratio in the patient group was found to be significantly higher compared to the control group (*p* = 0.009). The patient and control groups, however, did not exhibit a significant difference in Cu levels. Nevertheless, the Cu/Zn value (AUC: 0.613 and *p* = 0.007) emerged as a reliable predictor of CTE diagnosis.

The multifactorial etiology of CTE, which encompasses inflammatory, infectious, and nutritional deficiencies, necessitates the identification of a tangible biomarker to aid in its diagnosis. In this regard, our research findings have demonstrated that the Cu/Zn ratio is a reliable biomarker for CTE diagnosis, in consonance with existing literature. Our study further underscores the importance of the Cu/Zn ratio as an objective interpretation tool for CTE, given its subjective diagnostic grounds. Notably, our results reveal an increase in the Cu/Zn ratio among CTE patients, which suggests an augmented risk of the disease. Our study thus lays the foundation for extensive research on the use of the Cu/Zn ratio as a diagnostic tool for CTE.

Se is a dietary trace element that has recently garnered increased attention for its antineoplastic and anti‐inflammatory properties. In order to elucidate the role of Se in hair growth, the effects of Se imbalance on hair follicles were investigated in a C57BL/6 mouse model. Specifically, the study examined the effects of diets containing excess, adequate, or deficient Se on hair follicles. As a result, mice receiving excess or deficient Se displayed symptoms of poliosis and alopecia. Additional examinations of skin biopsies from alopecia patches revealed epidermal atrophy and increased telogen hair follicles. These findings highlight the potential role of Se in hair growth and suggest that Se balance is crucial for maintaining healthy hair follicles.^27^

Our study found that the average Se value of patients with CTE was significantly higher than that of the control group, but both groups fell within the reference range of the research kit. This suggests that the optimal serum level and therapeutic window of Se for hair disorders may be quite narrow. Based on our findings, we concluded that Se levels have diagnostic value in identifying CTE, and that elevated serum Se levels may be a risk factor for developing CTE.

Upon conducting an extensive review of the current literature, it has been found that no studies have been conducted on the comparison of Se levels in CTE patients with the control, making our study the first of its kind in this respect. Although several studies have investigated Se levels in relation to AA, it is widely acknowledged that there is insufficient data to determine the association between alopecia and serum Se levels. As such, it is clear that further research is warranted to explore this subject in greater depth.^28^, ^29^

The current study is subject to certain limitations that warrant consideration when interpreting the findings. The diagnosis of CTE was established on the basis of a clinical examination, hair pull test, dermoscopic findings, and exclusion of other diseases. However, due to the invasive nature of the procedure, trichogram analysis could not be performed. Furthermore, it is noteworthy that the study was conducted exclusively at a single center, which may restrict the generalizability of the results.

In conclusion, the optimal approach to treating female patients with CTE entails obtaining a detailed anamnesis concerning the etiology, conducting a comprehensive physical examination, and requesting a blood test to identify potential underlying conditions. Our study found no significant disparity between the incidence of iron, vitamin B12, and vitamin D deficiencies among young women diagnosed with CTE and the control group. Thus, it is imperative to conduct a thorough evaluation of female patients presenting with CTE symptoms to identify any underlying deficiencies that may be contributing to their condition. It has been observed that among the elements that are commonly associated with TE, including biotin, Zn, Cu, and Se, only the levels of Zn were found to be significantly lower in patients. Conversely, the levels of Se were found to be significantly higher. This highlights the importance of an individualized approach when treating patients, rather than blindly prescribing multivitamins and mineral supplements to each patient.

## AUTHOR CONTRIBUTIONS

İ.N.D.T., A.K.T., S.S., R.D. and G.G. performed the research. İ.N.D.T., S.S. designed the research study. İ.N.D.T, S.S., R.D. and G.G. contributed essential reagents or tools. İ.N.D.T., A.K.T. and G.G. analysed the data. İ.N.D.T and A.K.T wrote the paper.

## FUNDING INFORMATION

This study was supported by the AFSU Faculty of Medicine Scientific Research Projects Unit (Project no. 22.TUS.009) and approved by the AFSU Non‐Interventional Clinical Research Ethics Committee (Number: 2022/9 Date: 05.08.2022).

## CONFLICT OF INTEREST STATEMENT

The authors declares that there is no conflict of interest regarding the publication of this paper.

## ETHICS STATEMENT

All procedures performed in studies involving human participants were in accordance with the ethical standards of the institutional and/or national research committee and with the 1964 Helsinki declaration and its later amendments or comparable ethical standards.

## CONSENT

The authors give their consent for the publication of identifiable details, which can include photograph(s) and/or videos and/or case history and/or details within the text to be published in the above Journal. Written informed consent forms were obtained from the participants.

## Data Availability

The data that support the findings of this study are available from the corresponding author upon reasonable request.
